# Relative Importance of Positive Aging Dimensions Among Latino Older Adults and Service Providers

**DOI:** 10.1093/geroni/igaa057.1100

**Published:** 2020-12-16

**Authors:** Jennifer Smith, Cate O’Brien, Joseph Bihary

**Affiliations:** Mather Institute, Evanston, Illinois, United States

## Abstract

The variation in Latino older adults’ conceptualizations of positive aging across studies suggests greater attention should be paid to within-group factors. The purpose of the current study was to identify which factors are important to positive aging from the perspective of Latino older adults, and whether the importance of these factors varied based on participant characteristics. A second aim of this study was to examine whether there are differences in views of successful aging between Latino older adults and service providers who support aging Latinos. The current study was conducted as part of a broader research project investigating Latino older adults’ perceptions of positive aging. Latino older adults (n = 93) and aging services providers (n = 45) rated the importance of a series of statements related to positive aging. Mixed-methods analysis of the statements identified nine distinct dimensions (Positive Outlook, Spirituality/Religion, Healthy Behaviors, Independence, Self-Care, Support for Others, Social Support, Leisure Activities, and Adaptability). Latino older adults rated Positive Outlook and Spirituality highest on importance, and ratings differed based on gender and other individual difference characteristics. For example, men placed greater relative importance on Independence and Support for Others compared to women, and younger participants rated Independence higher on importance compared to older participants. In addition, Latino older adults (vs. providers) placed greater importance on all aspects of positive aging, with greatest mean differences related to providing Support for Others and Spirituality. These findings have implications for wellness programs for Latino older adults and training for service providers.

